# Acute hydrocephalus secondary to traumatic perimesencephalic pneumocephalus: A case report

**DOI:** 10.1097/MD.0000000000018654

**Published:** 2020-01-31

**Authors:** Guichen Li, Guangming Wang, Tengfei Luan, Kun Hou, Jinlu Yu

**Affiliations:** a Department of Neurology; b Department of Neurosurgery, The First Hospital of Jilin University, Changchun, Jilin, China.

**Keywords:** acute hydrocephalus, pneumocephalus, traumatic subarachnoid hemorrhage

## Abstract

**Introduction:**

Acute hydrocephalus is a common complication of spontaneous or traumatic intracranial bleeding with extensive subarachnoid hemorrhage (SAH) or ventricular extension. However, it has never been reported to be secondary to pneumocephalus.

**Patient concerns:**

A 32-year-old man was admitted following a motorcycle accident. Head computed tomography (CT) performed right after the accident revealed a skull base fracture and mild perimesencephalic SAH. Three days later, repeated CT revealed delayed perimesencephalic pneumocephalus and an evident enlargement of the ventricular system.

**Diagnosis:**

The patient was diagnosed with acute obstructive hydrocephalus, which was secondary to pneumocephalus and traumatic SAH.

**Interventions:**

The patient was treated with temporary external ventricular drainage (EVD).

**Outcomes:**

The patient experienced an unremarkable recovery process. At follow-up 3 months later, he showed no recurrence of the hydrocephalus and the score of Glasgow Outcome Scale was 5.

**Conclusion:**

Transient mechanical obstruction of CSF circulation and disturbance of CSF physiology might conjointly lead to the acute obstructive hydrocephalus.

## 1 Introduction

Acute hydrocephalus is a common complication of spontaneous or traumatic intracranial bleeding with extensive subarachnoid hemorrhage (SAH) or ventricular extension.^[1,2]^ Pneumocephalus is often encountered in patients with open craniocerebral injuries or skull base fractures. A gradual accumulation of a large amount of air inside the cranium may lead to increased intracranial pressure and mass effect (tension pneumocephalus).^[3]^ In addition to headache, pneumocephalus may cause confusion, lethargy, hemiparesis, and hemiplegia.^[4]^ Hardly could we ever imagine that acute hydrocephalus occurs after traumatic pneumocephalus. In this report, we present a rare case of acute hydrocephalus secondary to traumatic mild SAH and delayed perimesencephalic pneumocephalus. To the best of our knowledge, no previous similar case was reported.

## 2 Case report

**Figure 1:**
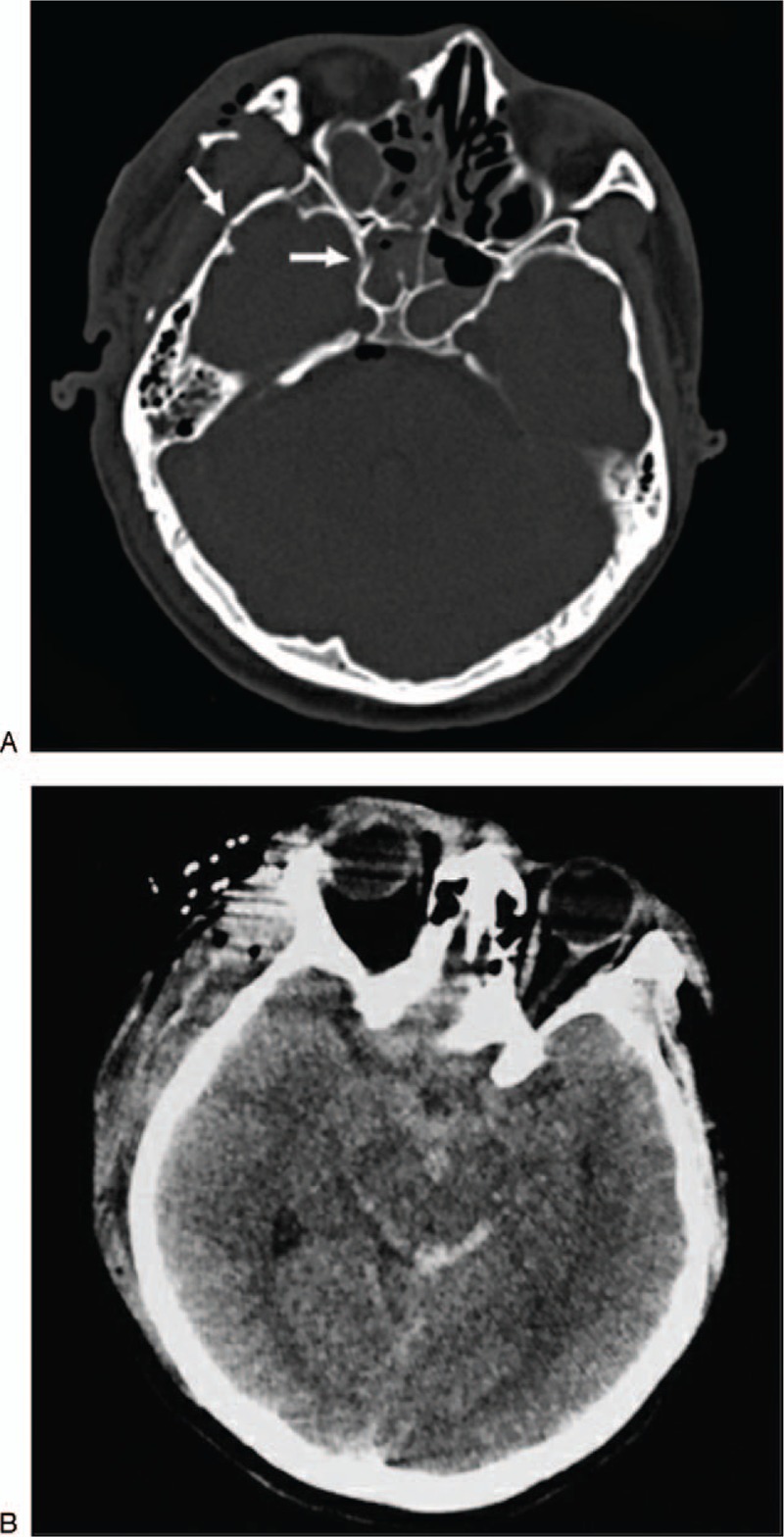
(A and B) Computed tomography on admission shows fracture of the temporal bone (arrow) and sphenoid sinus (arrow) and subtle perimesencephalic subarachnoid hemorrhage.

**Figure 2:**
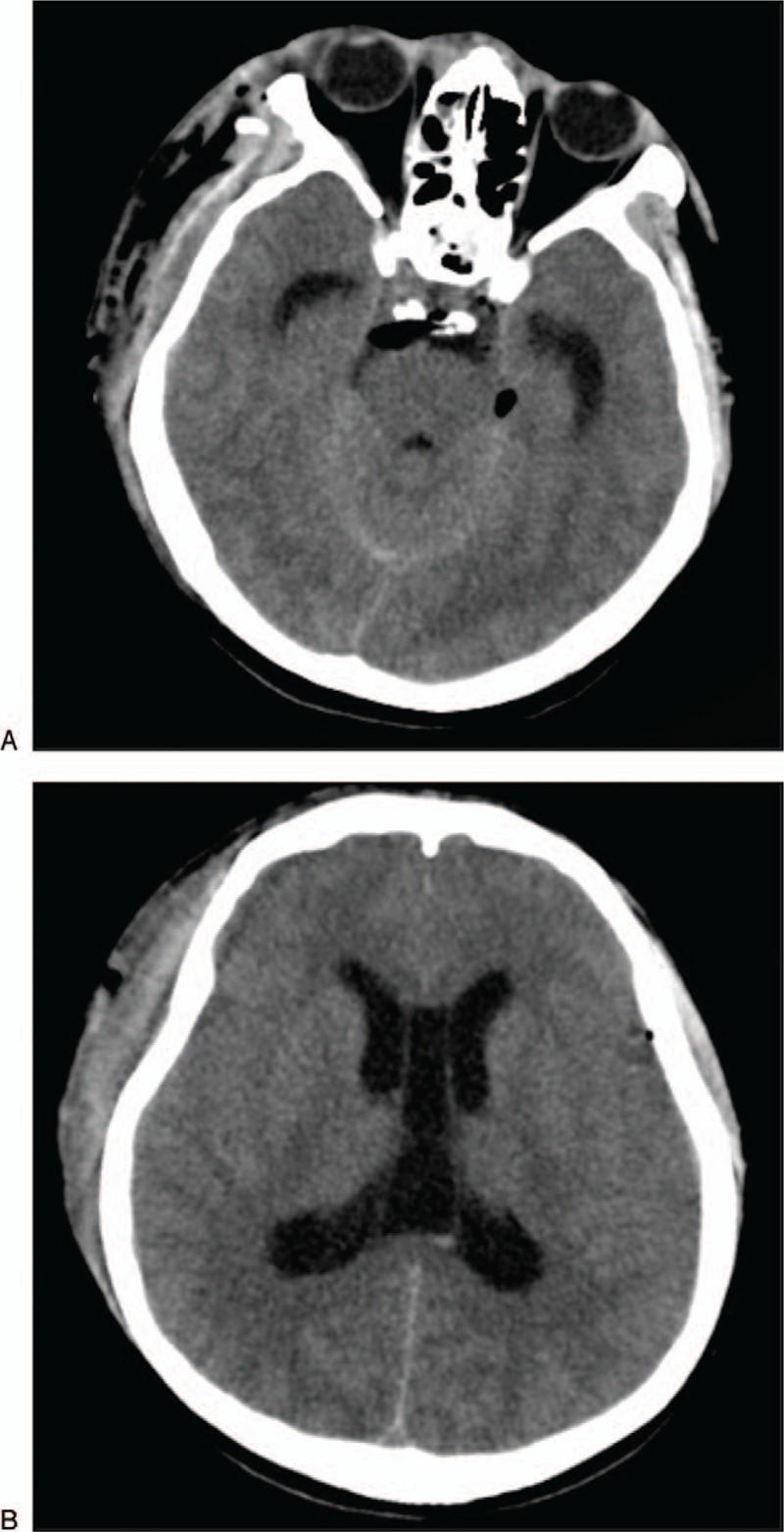
(A and B) Computed tomography performed 3 days later shows perimesencephalic pneumocephalus and evident enlargement of the ventricular system. The bilateral cistern ambiens vanish.

**Figure 3:**
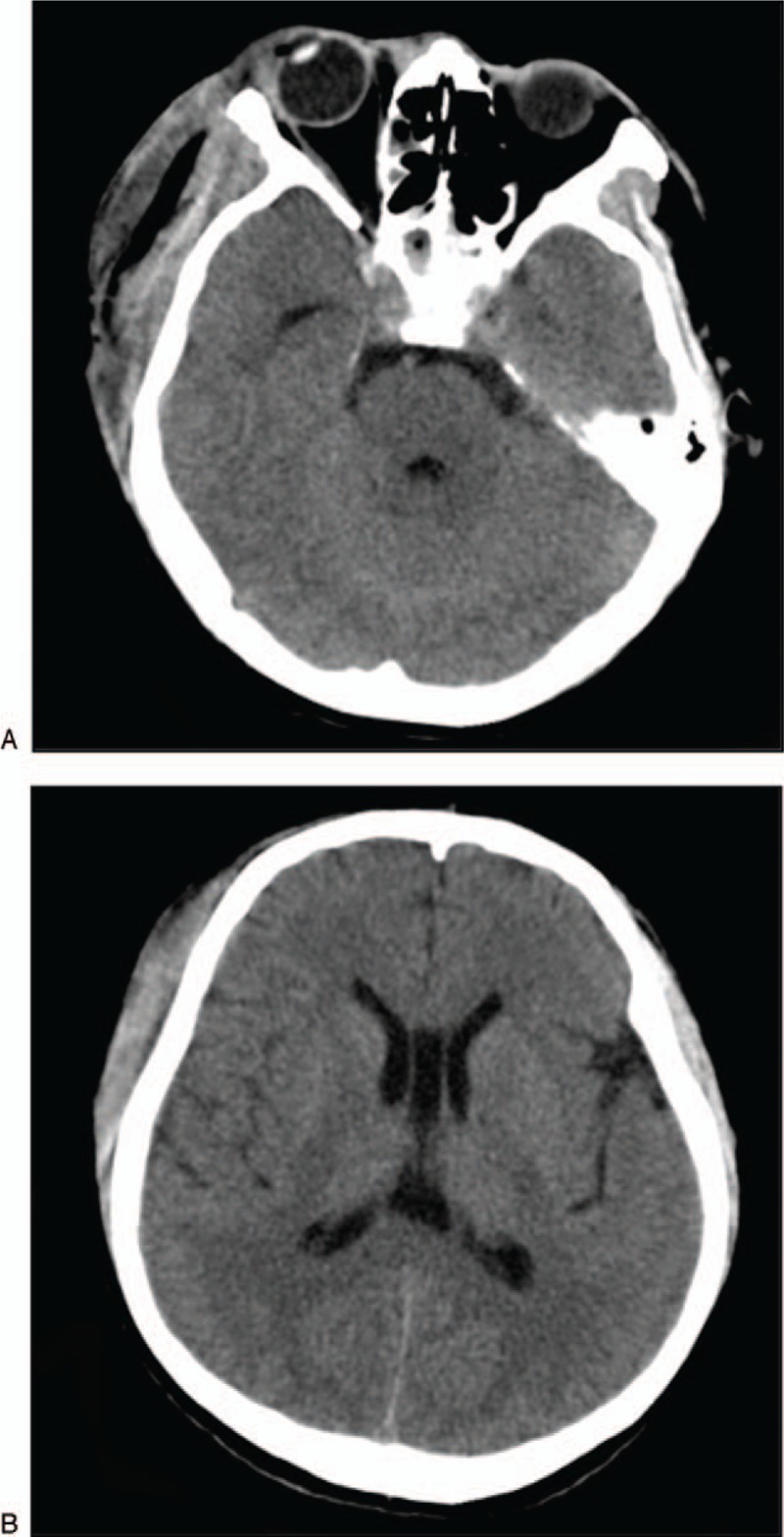
(A and B) Computed tomography shows the ventricle size returns to normal.

A 32-year-old man was admitted to the local hospital following a motorcycle accident. Head computed tomography (CT) performed right after the accident revealed a skull base fracture and mild perimesencephalic SAH (Fig. 1). He was alert and could obey commands correctly on admission. Physical examination was unremarkable except for right cerebral spinal fluid (CSF) otorrhea. He was managed with strict bed rest and prophylactic antibiotics, with the anticipation of spontaneous resolution of CSF otorrhea. Three days later, his mental status began to decline progressively. Head CT revealed delayed perimesencephalic pneumocephalus and an evident enlargement of the ventricular system (Fig. 2). A diagnosis of acute hydrocephalus was achieved. He was transferred to our institution for further treatment. On admission, he was comatose and had a GCS (Glasgow Coma Scale) score of 9 (M5V2E2). A temporary external ventricular drainage (EVD) was performed via the frontal horn of the right lateral ventricle on the fourth day after accident. His GCS score returned to 15 ten hours after EVD placement. The CSF investigations were not in favor of intracranial infection. The pneumocephalus and CSF otorrhea gradually resolved and the EVD was removed 3 days postoperatively. He experienced an unremarkable recovery process. At follow-up 3 months later, he showed no recurrence of the hydrocephalus (Fig. 3) and the score of Glasgow Outcome Scale was 5.

## 3 Ethic statement

Being a Case Report with written consent, our institution does not require formal Ethical Approval. Written informed consent was obtained from the patient for publication of this case report.

## 4 Discussion

Acute hydrocephalus refers to rapid ventricular enlargement and is commonly encountered after spontaneous or traumatic intracranial bleeding, CSF infection, or intracranial occupying lesions.^[2]^ However, it has never been reported in patients with traumatic pneumocephalus. In a report by Lee et al, a case of delayed shunt-dependent communicating hydrocephalus was reported 2 months after surgery for tension pneumocephalus.^[5]^ However, the patient is different from ours in that our patient developed delayed hydrocephalus concurrently with the development of pneumocephalus. In addition, the hydrocephalus of this case was transient and no permanent shunting was needed. Therefore, they might have different pathophysiological mechanisms. The possible explanations that involved in the development of acute hydrocephalus in this patient might be as follows.

First, obstruction of CSF circulation caused by perimesencephalic pneumocephalus. Our deduction was mainly based on the following facts. In contrast, acute hydrocephalus was previously reported following pneumoencephalography, which was thought to be obstruction of CSF circulation.^[6]^ In contrast, the patient did not develop hydrocephalus at the acute stage of head injury but 3 days later with the concurrence of perimesencephalic pneumocephalus. The hydrocephalus did not recur after resolution of the pneumocephalus and withdrawal of the EVD. CSF upward circulation to the supratentorial basal cisterns might be blocked by entrapped air at the perimesencephalic cistern.

Secondly, relatively hyperosmotic environment caused by SAH. From the traditional and widely accepted viewpoint, CSF is actively secreted by the choroid plexuses in the ventricles and circulates unidirectional along the ventricular system toward the cortical subarachnoid space and is ultimately absorbed into the venous sinuses through arachnoid villi. However, with some new findings in experimental investigations on animals and humans, a new hypothesis of CSF physiology and hydrocephalus development was proposed which was named Bulat-Klarica—Orešković hypothesis.^[7,8]^ According to this hypothesis, cerebral interstitial fluid (ISF) as well as CSF, is formed by water (constituting 99% of CSF) filtration across arterial capillary walls throughout the entire central nervous system (CNS). A continuous turnover of water takes place in microvessels between the plasma and ISF/CSF. The exchange of water inside the CNS is influenced by osmotic and hydrostatic forces. The water exchange between the entire ventricular system and the ISF depends on changes in fluid osmotic and hydrostatic pressures in different CNS compartments. That is to say, the volume of CSF is regulated by the osmotic and hydrostatic difference between the CSF/ISF and microvessels. In this patient, perimesencephalic SAH and its degradation products might cause local inflammatory responses and relatively hyperosmotic environment and lead to water movement from the ISF and microvessels to the ventricular system. Although this theory of CSF formation has not been widely accepted and could not overturn the traditional theory at present, it could at least shed some light on the formation of hydrocephalus in this case.

## 5 Conclusions

We report a rare case of acute hydrocephalus secondary to traumatic SAH and delayed perimesencephalic pneumocephalus. The underlying mechanism might be transient mechanical obstruction of CSF circulation and disturbance of CSF physiology.

## Author contributions

**Conceptualization:** Guangming Wang, Kun Hou, Jinlu Yu.

**Data curation:** Guichen Li.

**Project administration:** Jinlu Yu.

**Writing – original draft:** Guichen Li, Tengfei Luan.

**Writing – review & editing:** Kun Hou, Jinlu Yu.
